# Neurological research and practice: the first milestone has been reached

**DOI:** 10.1186/s42466-021-00101-8

**Published:** 2021-01-13

**Authors:** Werner Hacke

**Affiliations:** Department of Neurology, University of Heidelberg, Im Neuenheimer Feld 400, 69120 Heidelberg, Germany

Almost 2 years ago, on February 28, 2019, the first set of articles appeared in the new peer-reviewed open access online-only scientific Journal “Neurological Research and Practice” (NRP). The journal is published jointly by Biomed Central (BMC, a division of Springer-Nature Publishers) and the German Neurological Society (Deutsche Gesellschaft für Neurologie, DGN, Berlin). NRP is the official journal of the German Neurological Society (Deutsche Gesellschaft für Neurologie (DGN)), one of the largest national neurological societies worldwide with more than 10,000 members. NRP was created by the leadership of the DGN as an international English language publication with a broad thematic scope reflecting all clinical, translational, and basic research aspects of neurology and neuroscience [1]. Currently the society bares all publication cost. There are no fees for authors.

In the first year, we published 41 articles. We were pleased by the quality of the reviews (12) and research articles (16), the innovation of the clinical trial protocols, and the reliability of Guidelines and Standard operating procedures [2]. This development continued in the second year of publications: Over 50 articles were published (Table 1). We noticed a sharp increase of letters, about half of the COVID-19 related, while the number of reviews and Research articles remained stable. Overall, we had an acceptance rate of 50%, with the number of letters rejected in the order of 70%.

**Table 1:** Submissions, article types, and citations 2019 and 2020

Year	Reviews	Research Articles	Clin. Trial Prot.	Guidelines	SOPs	Letters	Editorials	Total
2019	12	16	4	4	3	1	1	41
2020	11	14	6	5	1	12	1	50
Citations	33	54	7	7	3	9	1	113

In total, more than 120 citations have been counted by now. Table 2 lists the five articles [3–7] with the highest citations (2 research articles, 2 reviews, and a letter published in 2020. It is important to note that clinical trial protocols, guidelines, and also SOPs are frequently cited. In our opinion, this underlines the importance of these article types. The far majority of the letters were published in 2020 and several have already been cited.

**Table 2:** Most cited NRP-articles in 2019 and 2020

Author	Title	Reference	Nr. of citations	Article type
Dziewas et al. [3]	Safety and clinical impact of FEES	*Neurological Research and Practice* **2019** 1:16	16	Research
Weber et al. [4]	Distribution and evolution of acute interventional ischemic stroke treatment in Germany from 2010 to 2016	*Neurological Research and Practice* **2019** 1:4	21	Research
Mokli et al. [5]	Computer-aided imaging analysis in acute ischemic stroke – background and clinical applications	*Neurological Research and Practice* **2019** 1:23	7	Review
Göttle et al. [6]	An unmet clinical need: roads to remyelination in MS	*Neurological Research and Practice* **2019** 1:21	5	Review
Lampe et al. [7]	Guillain-Barré syndrome and SARS-CoV-2	*Neurological Research and Practice* **2020** 2.19	5	Letter

Access rates also show how much interest some of our articles have received. A review on the “resumption of oral anticoagulation after intracerebral hemorrhage” has already received almost 10,000 accesses [8]. Other highly accessed articles are found in Table 3 [9–11]. Three of them were published in 2020. The publication highlights include the first-ever guideline on “Neurological manifestations of COVID 19” [9], the results of a national registry of fiberoptic endoscopic evaluation of swallowing [3], the worldwide first documentation of the population-wide uptake of mechanical thrombectomy [4], and a review of antibody associated movement disorders [11].

**Table 3:** Most accessed NRP-articles in 2019 and 2020

Author	Title	Reference	Nr .of accesses	Article type
Sembill et al. [8]	Resumption of oral anticoagulation after spontaneous intracerebral hemorrhage	*Neurological Research and Practice* **2019** 1:12	9815	Review
Berlit et al. [9]	“Neurological manifestations of COVID-19” - guideline of the German society of neurology	*Neurological Research and Practice* **2020** 2:51	6955	Guideline
Busetto et al. [10]	How to use and assess qualitative research methods	*Neurological Research and Practice* **2020** 2:14	5010	Review
Gövert et al. [11]	Antibody-related movement disorders – a comprehensive review of phenotype-autoantibody correlations and a guide to testing	*Neurological Research and Practice* **2020** 2:6	4359	Review
Dziewas et al. [3]	Safety and clinical impact of FEES	*Neurological Research and Practice* **2019** 1:16	4186	Research

In December 2020, NRP was included into Pubmed in a record-breaking time of less than 2 years after the publication of the first articles.This is a great breakthrough and the first milestone on our way to a widely accepted international scientific journal. We believe that this listing will lead to an increasing number of article submissions. We hope that our publications will be cited even more frequently, which will lead eventually to our first impact factor in a short time.

Our publication pipeline continues to grow, and the number of international submissions is increasing. So far, we have received 55 submissions from authors outside of Germany. We will continue to invite reviews on new developments and perspectives, and we welcome the submission of research articles. Several clinical trial protocols are in preparation, and we have lists of upcoming standard operating procedures and guidelines to be published within the next 12 months.

The Editorial Board has been a great source of support for establishing NRP, and we will have the first rotation of our Editorial Board in 2021. We will announce Section Editors for key areas in neurology such as neuroimmunology and neurooncology, neurodegeneration and movement disorders, stroke, interventional neurology and critical care, pediatric neurology, cognitive neurology, and functional imaging, and neuromuscular disorders.

As the founding editor of NRP I am grateful for the ever increasing support by the leadership of the German Neurological Society represented by our current President Professor Christine Klein, Past President Professor Gereon Fink, Incoming President Christian Gerloff, the Secretary Professor Peter Berlit, and the Chief Administrative Officer Dr. Thomas Thiekötter. I express my gratitude to our partners at the publisher’s office, namely Emily Jones and Cecille Calusa for their continuous support, to all members of the Editorial Board, to the authors for submitting their articles, and to the reviewers for their most important contributions. They all make the first years of NRP so successful.

Please continue to support us to make NRP a well-received and influential voice of German neurology publishing content with international relevance.
